# Henoch-Schönlein purpura in the third trimester of pregnancy

**DOI:** 10.11613/BM.2018.010801

**Published:** 2018-01-10

**Authors:** Ivka Djakovic, Drazan Butorac, Zeljko Vucicevic, Vesna Kosec, Andrea Tesija Kuna, Liborija Lugović-Mihić

**Affiliations:** 1 Department of Gynaecology and Obstetrics, Sestre milosrdnice University Hospital Center, Zagreb, Croatia; 2 Department of Internal Medicine, Intensive Care Unit, Sestre milosrdnice University Hospital Center, Zagreb, Croatia.; 3 University Department of Chemistry, Medical School Sestre milosrdnice University Hospital Center, Zagreb, Croatia; 4 Clinical Department of Dermatovenereology, Sestre milosrdnice University Hospital Center, Zagreb, Croatia

**Keywords:** Henoch-Schönlein purpura, leukocytoclastic vasculitis, pregnancy, glomerulonephritis, preeclampsia

## Abstract

Henoch-Schönlein purpura (HSP) is an IgA-mediated small vessels’ vasculitis that affects the skin, intestines and kidneys. Pregnancy has been reported as an exacerbating factor. We present the case of a 24-year-old *primigravida* with HSP that occurred in the third trimester and lasted up to the end of the successful delivery. She had pruritic maculopapular exanthema on her legs. Biopsy of a cutaneous lesion was performed for histopathologic features and direct immunofluorescence (DIF) for the presence of perivascular IgA deposition. Histopathology of the cutaneous lesion confirmed leukocytoclastic vasculitis. A DIF examination of the skin lesion confirmed deposits of fibrinogen in the small blood vessel walls. Six weeks following delivery, the skin lesions almost completely disappeared. Control laboratory findings were normal. This case of HSP might have been primarily associated with a preceding respiratory infection but this should first be carefully investigated due to a possible severe immunological disease in the patient’s background requiring special attention since nephrotic symptoms may occur.

## Introduction

Henoch-Schönlein purpura (HSP) is a subtype of acute leukocytoclastic vasculitis (LCV) characterized by immunoglobulin (Ig) A immune complex deposits within the small blood vessel walls of the skin and other organs (*1*). The most common clinical manifestations include maculopapular rash on the lower extremities and buttocks, fever, large joints arthralgia, gastrointestinal disorders and glomerulonephritis which occurs much more frequently in adults (*1*-*4*). Incidence is higher in children and males (*3*, *5*, *6*). Streptococcal respiratory tract infections or other infectious agents precede HSP, but certain foods, drugs, insect bites and vaccines may also play a role (*1*).

Diagnosis of HSP is based on clinical symptoms and confirmed by biopsy and histopathological proof of LCV (*2*). The disease usually retreats after a month but there is a possibility of recurrence (*7*-*9*). Sometimes it is necessary to treat HSP with corticosteroids and in the case of the kidney disorders immunosuppressive therapy is necessary (*4*, *9*-*11*).

HSP is rarely described in pregnancy (*12*-*15*). The occurrence of HSP in pregnancy can affect the mother and the foetus. Especially dangerous situation is when kidneys are affected and uraemia occurs (*16*-*20*). We present a case of HSP that occurred at the beginning of the third trimester of pregnancy and lasted up to the end of the successful delivery.

## Case report

### Patient information and clinical findings

**Figure 1:**
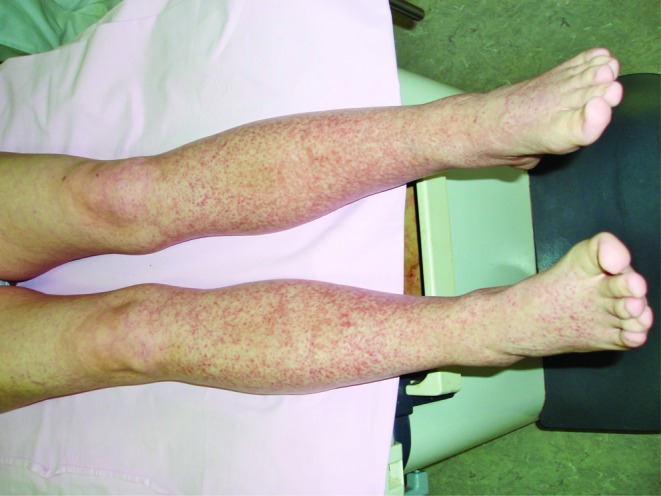
Purpuric lesions of the patient’s shins

A 24-year-old *primigravida* in the 35th week of pregnancy was admitted to our obstetric department for lower extremities pruritic maculopapular exanthema that had appeared a day before (Figure 1). The patient had no abdominal pain and no arthralgias. She had congenital right forearm aplasia with no family history of autoimmune diseases. An amniocentesis performed previously revealed a normal female karyogram. Obstetric findings at admission were normal. No allergies were recorded. She had gained 8 kilograms and continued to smoke during pregnancy. The patient was recovering from a recent respiratory infection. According to anamnestic data, it is rather likely that our patient had experienced similar symptoms two years earlier that spontaneously disappeared.

Body temperature, blood pressure, and routine laboratory tests were normal with the exception of an elevated erythrocyte sedimentation rate (ESR) of 45 mm/3.6 ks. Differential laboratory evaluation included the determination of antistreptolysin O (ASO) titre, IgG, IgA and IgM, C3 and C4 component of complement, rheumatoid factor (RF) performed by immunoturbidimetric method on AU2700 analyser (Beckman Coulter Inc, Brea, USA) and anti-staphylolysin-α-hemolysin (AStaL) titre using an agglutination assay (Siemens Healthcare Diagnostics Products GmbH, Marburg, Germany). Furthermore, antinuclear antibodies (ANA) were determined using the “gold standard” method, indirect immunofluorescence on human epithelioma type 2 cells (HEp-2) (commercial assay, Euroimmun AG, Luebeck, Germany). Anti-neutrophil cytoplasmic antibodies (ANCA) were determined by indirect immunofluorescence using triple substrates including ethanol fixed granulocytes as the primary substrate, and formalin-fixed granulocytes and HEp-2 cell substrates as auxiliary substrates for fluorescence differentiation originated from ANCA and ANA, which can mimic perinuclear ANCA (commercial assay, Euroimmun AG, Luebeck, Germany). Antibodies to extractible nuclear antigens (ENA) were determined by a semiquantitative commercial enzyme-linked immunosorbent assay (ELISA) (Euroimmun AG, Luebeck, Germany) and included the following antigens: SS-A/Ro, SS-B/La, Scl-70, Sm, RNP/Sm and Jo-1. The manufacturer declared intra- and inter-assay coefficients of variation (CVs) for individual antibodies as follows: 3.0%, 3.4%; 3.8%, 5.2%; 4.1%, 4.6%; 2.3%, 3.6%; 3.6%, 4.3%; 2.7% and 3.2%, respectively. Commercial ELISA tests were used for quantitative determination of antibodies to dsDNA (Orgentec Diagnostika GmbH, Mainz, Germany, with declared intra- and inter-assay CVs of up to 6.4% and 12.4%, respectively) and antibodies to ANCA specific antigens: proteinase 3 (Pr3) and myeloperoxidase (MPO) (both Euroimmun AG, Luebeck, Germany, with declared intra- and inter-assay CVs of up to 4.1% and 11.2% for anti-Pr3, and 4.4% and 5.1% for anti-MPO, respectively). All tests were performed according to the manufacturer’s instructions. The patient’s laboratory test results are given in Table 1.

**Table 1:** The patient’s laboratory test results

**Test (unit)**	**Result**	**Reference value**
IgG (g/L)	18.0	7.0 – 16.0
RF (IU/mL)	88.8	≤ 14.0
ANA (titre)	Speckled fluorescence (1:320)	< 1:80
Anti-SS-A/Ro (U/mL)	5.8	Negative: < 1.0 Low positive: 1.0 – 2.0 Positive: 2.0 – 5.0 Highly positive: > 5.0
IgG – immunoglobulin G. RF - rheumatoid factor. ANA - antinuclear antibodies. Anti-SS-A (Ro) - antibodies to SS-A/Ro antigen.		

Biopsy of a cutaneous lesion was performed for the analysis of histopathologic features and direct immunofluorescence (DIF) for detection of the presence of perivascular IgA deposition. The histopathology results of the cutaneous lesion revealed leukocytoclastic vasculitis. Skin biopsies of the lesioned tissue from the patient were sent for DIF, which was performed by standard method (*21*). The biopsies were transported in phosphate buffer saline (PBS) (pH 7.2), frozen in a cryostat and sectioned at 6 μm. Sections were incubated with fluorescein isothiocyanate (FITC) conjugated, Fc-specific F (ab) 2 antisera directed against IgG (1:15 diluted), IgA (1:10 diluted), IgM (1:20 diluted), complement C3 (1:10) and fibrinogen (1:10) (Dako, Copenhagen, Denmark). Subsequently, specimens were incubated in a moist chamber at room temperature for 30 min, and excess antibodies were washed off with PBS three times. The specimens were analysed with fluorescence microscope. A specimen was considered to be positive if deposits of one or more immune-reactants were found in the walls of one or more vessels. Direct immunofluorescence examination of skin lesion confirmed deposits of fibrinogen in the small blood vessel walls.

Cervical smears were normal. Hepatitis B, C and HIV tests were negative. Nasal and throat smears were clean. The cytology of a peripheral blood smear was indicative of toxic granules in granulocytes. Ophthalmic and cardiac examinations were normal.

### Therapeutic intervention

There were no signs of visceral disease and no need for systemic corticosteroid therapy. The patient was discharged after one week and advised to avoid salicylates, penicillin, non-steroid anti-inflammatory drugs, and spicy foods.

### Follow-up and outcomes

In the last month of pregnancy the patient returned for a follow-up with a dermatologist, internist and obstetrician as an outpatient. The regression and spreading of the skin lesion interchanged during that period. There were no clinical or laboratory signs of arthralgias, renal failure or gastrointestinal bleeding.

The patient was hospitalized in the 40th week of pregnancy for planned delivery. Ultrasound and cardiotocographic records were normal. In the 41st week of pregnancy she developed regular contractions and gave birth to a healthy, new-born female weighing 3210 g and 49 cm in length. Her Apgar scores were 10. The third stage of labour went without complications. Six weeks following delivery, the skin lesions almost completely disappeared. Control laboratory findings were normal. The patient signed an informed consent.

## Discussion

Henoch-Schönlein purpura is rarely described in pregnancy (*12*-*15*). A preceding respiratory infection could have been the trigger for an HSP episode in our patient.

Pregnant women with previous episodes of HSP have an increased risk of complications in pregnancy, especially proteinuria and hypertension, and should be closely monitored (*12*, *13*). Obstetrical complications like preeclampsia require early diagnosis and treatment, especially if serious renal failure or HELLP syndrome (a variant of preeclampsia accompanied with haemolysis, elevated liver enzymes and low platelet count) are about to develop. Occasionally, in the very beginning, this condition is hardly distinguishable from HSP symptoms (*15*). The retrospective study of Ronkainen *et al.* showed that the pregnancies of 70% of women, who suffered HSP in childhood, were complicated by hypertension and/or proteinuria. More than half of all the women with pregnancy complications also had serious renal complications (*22*).

HSP has no specific symptoms in pregnancy and can be more difficult to diagnose if other extra-cutaneous symptoms occur before the typical skin lesions appear, since hypertension, nephrotic syndrome, and preeclampsia may disguise these symptoms (*23*). Headaches and mild behaviour changes suggest central nervous system involvement in one-third of HSP patients (*16*). No similar symptoms were noticed in our patient.

Substantial factors that can precipitate an HSP relapse or recurrence are persistent infective foci, particularly chronic tonsillitis (*5*). Chronic infective foci must be carefully searched for in order to eliminate them, if at all possible. In our patient, no evidence of chronic infection was found.

Elevated concentrations of rheumatoid factor, ANA and in particular of anti-SS-A/Ro require special attention since systemic lupus erythematosus and Sjogren’s syndrome may accompany LCV or HSP (*24*). Negative ANCA finding supports the diagnosis of HSP in differentiating ANCA-associated small vessel vasculitis (SVV) from immune complex SVV, which includes HSP (*25*).

Direct immunofluorescence for tissue-bound autoantibodies and other components provide a useful parameter for the diagnosis of autoimmune disorders, helping in the classification of histologically similar conditions. Thus, a positive DIF test for immune complexes in the investigated tissue enables higher accuracy in the diagnostic process (*26*).

In HSP, IgA deposits are found in capillaries and postcapillary venules of the skin. Immunoglobulin M, fibrinogen and C3 are found less often, like in our case. There is a correlation between duration of the skin changes and a positive DIF test. According to Larsen *et al.*, sensitivity and specificity of vascular IgA for the diagnosis of HSP is 0.86 and 0.84, respectively, confirming that vascular IgA is nonspecific. The utility of immunofluorescence vasculitis studies are influenced by clinical presentation and the clinician’s level of suspicion of HSP (*27*).

It has been found that various conditions present in neonatal lupus are linked with anti-SS-A/Ro antibodies. However, presence of anti-SS-A/Ro antibodies has no influence on pregnancy outcome if followed appropriately in a specialized institution. For instance, congenital heart block in cases of Anti-SS-A/Ro positive women is 1 – 2%, neonatal lupus rash is found in 10 - 20% and abnormalities in laboratory tests in 27% of new-borns (*28*). In our case, the mother and the new-born were directed to the attention of the immunologist.

The placenta is a vascular labyrinth, therefore this dynamic of the foetal condition, its growth and development should be monitored (*18*, *23*). Mothers’ IgA antibodies are not present in foetal circulation due to the placental barrier and thus cannot cause foetal vasculitis. Histopathological analyses confirm this thesis (*23*, *29*). In our case, it seems that HSP has not affected the child’s early development, but it would be very interesting to investigate any disorder occurring later in the child’s life, especially those affecting the kidneys. Pregnancy does not have a great impact on the outcome of vasculitis, which is not the case with systemic lupus erythematosus. Therefore, a multidisciplinary follow-up is advisable (*30*).

It should be emphasized that pregnancy itself has been reported as a risk factor for HSP. In the available literature there is a case report of HSP recurrence in the 12th gestational week, following 19 years of remission. Therefore, pregnancy should be on the list of possible triggers for HSP in susceptible individuals (*31*).

When kidneys are involved, the efficiency of corticosteroids and immunosuppressants (azathioprine, cyclophosphamide) seems to be controversial. Diaminodiphenyl-sulfone (DDS) is an antibiotic described to be useful in various skin conditions and HSP, but could not be recommended for routine use (*23*). Plasmapheresis has been used in a number of patients, who had severe and crescentic disease and rapidly progressive renal failure, but its efficacy is uncertain and there are potential side effects. However, limited data suggest that plasmapheresis alone may be curative in some patients (*23*, *28*).

In conclusion, HSP in pregnancy may be triggered by usual risk factors, in most cases respiratory infections, but special attention must be given to resulting consequences that can jeopardize the mother and the pregnancy. When it does not resolve spontaneously, a multidisciplinary approach is recommended, especially regarding treatment, pre-term delivery and basic medical care for both mother and a child afterwards.
